# Prosocial behavior in toddlerhood and early childhood: Consistency across subtypes and over time

**DOI:** 10.3389/fpsyg.2023.950160

**Published:** 2023-02-23

**Authors:** Yael Paz, Maayan Davidov, Tal Orlitsky, Mor Hayut, Ronit Roth-Hanania, Carolyn Zahn-Waxler

**Affiliations:** ^1^The Paul Baerwald School of Social Work and Social Welfare, The Hebrew University of Jerusalem, Jerusalem, Israel; ^2^School of Behavioral Sciences, Academic College of Tel Aviv-Yaffo, Tel Aviv-Yaffo, Israel; ^3^Center for Healthy Minds and Department of Psychology, Center for Healthy Minds and Department of Psychology, University of Wisconsin–Madison, Madison, WI, United States

**Keywords:** prosocial behavior, childhood, compassion, individual differences, longitudinal study

## Abstract

**Introduction:**

Young children show their capacity for compassion and their desire to enhance the welfare of others in multiple ways. The present study sought to address gaps in knowledge regarding prosociality in the early years. Specifically, the study examined whether different subtypes of prosociality are interrelated, whether they are consistent over time, as well as the meaning of young children’s spontaneous versus cued prosocial behavior.

**Methods:**

In a longitudinal sample (*N* = 151), three subtypes of prosocial behavior—instrumental helping, compassionate helping (comforting), and sharing—were assessed using behavioral tasks in toddlerhood (18 months) and early childhood (36 months).

**Results:**

Consistent with hypothesis, partial convergence was found between the different prosociality subtypes at each age. There was also modest continuity over time, both within and across prosocial subtypes. Moreover, at both ages, when children helped or shared spontaneously, they also provided more assistance in the task. Children’s tendency to assist spontaneously was partially consistent across situations by early childhood.

**Discussion:**

The findings indicate that a moderately stable disposition toward prosociality is already evident during early ontogeny. Moreover, different subtypes of prosocial behavior are distinct yet interrelated in the early years, suggesting they have both common and unique underlying mechanisms. Lastly, young children’s spontaneous (versus cued) prosocial action appears to reflect both motivational and cognitive processes.

## 1. Introduction

Prosocial behavior, defined as benevolent acts toward others without direct benefit to the self (Eisenberg et al., 2006), is considered one of the cornerstones of a harmonious society and a testament to the human potential for compassion (Davidov et al., 2016). Prosociality is a multifaceted construct, encompassing a variety of ways in which children (or adults) can try to assist or further the needs of others (Dunfield et al., 2011; Brownell, 2013; Paulus, 2018). Prosocial behavior is seen early in development: By the second year of life, infants can already perform a variety of prosocial acts (Brownell, 2013; Paulus, 2018). Even during the first year, some infant behaviors may reflect simple prosocial actions (Liddle et al., 2015; Hammond et al., 2017). Moreover, young children seem eager to be helpful, seeking opportunities to assist others (Dahl, 2015) and taking pride in their helping (Hepach et al., 2017). There are considerable individual differences in early prosocial behavior: Whereas some children help or share frequently, others do so more rarely or selectively (Newton et al., 2016; Schachner et al., 2018). To gain further insight into the nature of these early individual differences, the present study examined their consistency across different subtypes of prosociality and across age. Our focus was, therefore, on concurrent and longitudinal associations, not on mean-level changes.

As we discuss below, examining these two forms of consistency in the same study can help shed light on fundamental questions regarding the nature and organization of early prosocial development. In particular, it can elucidate whether early prosocial behavior is trait-like or, rather, predominantly situational in nature, and if the former is true then whether young children’s prosocial disposition is broad or domain-specific (Penner et al., 2005; Kärtner et al., 2014; Knafo-Noam et al., 2015). As reviewed below, very few studies have examined consistency in prosocial behavior from toddlerhood to early childhood, particularly across different subtypes of prosociality (Kärtner et al., 2014; Paulus et al., 2015; Schachner et al., 2018). The present study addresses this gap.

### 1.1. Subtypes of prosociality and their interrelations

As noted above, prosociality is multidimensional, and encompasses different forms, or subtypes, of assisting or benefitting others. The most common classification of early prosocial behaviors is action-based, distinguishing between three types of prosocial acts: helping instrumentally, compassionate helping, and sharing (Dunfield et al., 2011; Brownell, 2013; Paulus, 2018). Instrumental helping refers to helping another individual complete an unattained pragmatic goal, such as getting an out-of-reach object or overcoming a physical obstacle (Warneken and Tomasello, 2006, 2007). The target of help has un unfulfilled goal, but typically does not express overt distress (if distress is expressed, the task is usually classified differently; Davidov et al., 2016; Newton et al., 2016). Compassionate helping (sometimes labeled “comforting”) includes helping or offering verbal or physical comfort to another in distress (Knafo et al., 2008; Newton et al., 2016). And sharing involves giving one’s own limited material resources to another individual (Brownell et al., 2013a; Newton et al., 2016).

There is ample evidence that these three subtypes of prosociality are distinct. First, although all three subtypes can be observed during the second year of life, their prevalence differs considerably: Instrumental helping is a common and frequent behavior in toddlerhood, whereas compassionate helping and sharing are much more rare, likely because these are more challenging behaviors for young children to enact, particularly toward strangers (Warneken and Tomasello, 2006; Knafo et al., 2008; Svetlova et al., 2010; Dunfield et al., 2011; Davidov et al., 2021). Second, these different forms of prosociality were shown to be linked with different antecedences and correlates (Paulus, 2018). For example, socio-cognitive factors such as joint attention and self-other differentiation were found to predict instrumental but not compassionate helping (Kärtner et al., 2014), whereas emotional talk (Drummond et al., 2014), parenting style, and child’s temperament (Schuhmacher et al., 2017) were all found to be more strongly associated with compassionate helping than with instrumental helping. Third, distinct neural pathways were also found to underlie instrumental and compassionate helping (Paulus et al., 2013).

Although the three subtypes of prosocial behavior are clearly distinct, they may still draw in part on common processes or mechanisms; for example, multiple subtypes may stem from the same motivation, and/or require some of the same cognitive abilities (Davidov et al., 2016). Such commonalities between subtypes of prosociality should be reflected by intercorrelations between them. There are, however, mixed findings in the literature regarding the interrelations among different subtypes of prosociality during the early years of life. Whereas some studies found no links between subtypes (Dunfield et al., 2011; Dunfield and Kuhlmeier, 2013; Kärtner et al., 2014; Paulus et al., 2015), others found modest convergence between them, when they were assessed concurrently (Sommerville et al., 2013; Brownell et al., 2013b; Newton et al., 2016; Schachner et al., 2018). Moreover, a study with 3.5-year-old twins found a positive correlation between their observed sharing and comforting behaviors, and this shared variance was accounted for in large part by common genetic factors (Knafo-Noam et al., 2018). And for 7-year-old twins, a general prosociality factor was identified, which was largely heritable and accounted for substantial portions of the variance in five different facets of prosociality, reported by mothers (Knafo-Noam et al., 2015); interestingly, in addition to the common factor, there were also unique genetic factors specific to each prosociality subtype. Thus, each subtype appears to have both common and unique mechanisms or features (Davidov et al., 2016).

Taken together, prior work points to a complex pattern, in which the three subtypes of prosociality are distinct on the one hand, yet often (but not always) converge partly on the other hand. Some of this inconsistency may be due to measurement issues, as even small differences in methodology can influence the degree and nature of prosocial behavior being assessed, and thus also the consistency of the child’s behavior across different measures (Thompson and Newton, 2013; Davidov et al., 2016). To shed further light on commonalities between subtypes of prosociality, additional systematic evidence is needed, particularly using multiple measures and with attention to issues of measurement error. The present study sought to address this gap, by examining the consistency of children’s prosocial responses both across subtypes and over time. We reasoned that this information could help distinguish between different possibilities regarding the nature of early prosocial development.

The first possibility is that early prosociality is not yet consistent or trait-like at all, but rather determined solely by situational and transient factors. If that is the case, then children’s prosocial responses should show little consistency both across subtypes and over time. Conversely, a second possibility is that even early in life prosocial behavior is trait-like, such that different forms of prosociality manifest, at least to some extent, the same core disposition or capabilities. Potential common mechanisms are other-oriented motivations (e.g., concern for others), and/or social-cognitive capabilities (the ability to understand what others need and how to assist them, e.g., theory of mind; Eisenberg et al., 2006, 2016). If that is true, then moderate consistency should be evident both across situations and over time; in particular, longitudinal links should be evident not only within, but also across, subtypes. The third possibility is that early prosociality does not reflect any general trait or disposition, nor is it merely situational, but rather it is domain-specific in nature (Kärtner et al., 2014). In this case, different subtypes of prosociality reflect distinct, separate sensitivities and capabilities, that develop largely independently of one another. If that is true, then longitudinal associations should be stronger within the same subtype of prosociality than across different subtypes. We therefore examined the associations among the three forms of prosociality at both 18 and 36 months.

### 1.2. Consistency across age in prosocial behavior

Longitudinal studies found positive associations between the same measure of prosocial behavior at different ages, suggesting modest consistency of individual differences over time (Eisenberg et al., 2015). For example, continuity was found from toddlerhood to early childhood in observed empathic concern for others in distress, a response which promotes compassionate helping (Knafo et al., 2008; Paz et al., 2022). Consistency was also found across early childhood for global questionnaire measures of prosociality, reported by parents (Girard et al., 2017; Jambon et al., 2019), and across middle childhood for parent-reported compassionate helping and cooperation, but not for observed sharing (Malti et al., 2016). In a study which assessed instrumental helping, compassionate helping, and sharing across early childhood (age 4.5 to 6 years), all three subtypes showed continuity of individual differences over this period (albeit fairly weakly for instrumental helping; Schachner et al., 2018). In another study, instrumental helping and compassionate helping (comforting) each showed modest continuity from 15 to 18 months (Kärtner et al., 2014; sharing was not assessed).

Nevertheless, systematic evidence is still needed regarding the consistency of individual differences in the three subtypes of prosociality between toddlerhood, when these behaviors emerge, to early childhood, when they are more prevalent and ingrained (Dahl, 2015). Given the vast, transformational changes that take place during this time period in children’s cognitive and social capabilities—including huge strides in language development, theory of mind, regulatory abilities, interactions with peers, and more—it is important to examine whether the tendency to act prosocially, by helping, sharing, and comforting, shows consistency across this period (Hay and Cook, 2007). The current study therefore examined continuity from 18 to 36 months.

Moreover, very little is known about the longitudinal links across different subtypes of prosociality (as opposed to within each type), which have often not been reported (Kärtner et al., 2014; Schachner et al., 2018). In one longitudinal study that examined links across subtypes of prosociality, instrumental helping at 18 month and compassionate helping at 24-months were not associated with each other or with sharing at 60-months (Paulus et al., 2015); however, this study did not assess longitudinal consistency within each subtype, making it hard to interpret the lack of associations across subtypes. Given the paucity of research, more work is needed to systematically examine the longitudinal consistency of prosociality, particularly across different subtypes of prosocial behavior. The present study addressed this gap.

### 1.3. Consistency in spontaneity of prosocial behaviors

As a secondary question, the present study also examined consistency in another aspect of children’s prosocial responding—its degree of spontaneity. Within each subtype, prosocial action can vary in the level of communication between the child and the needy other. This variability can be conceived of as a continuum, ranging from completely spontaneous assistance, evoked by the other’s need in the absence of any communication with the child (e.g., no eye contact, speech, gestures), through cued assistance, when the other hints to varying degrees that help is needed from the child or what help is wanted, and up to assistance given in response to very explicit cueing or direct requests, which can amount to compliance.

More explicit communication increases the likelihood of prosocial action (Svetlova et al., 2010), but it can also have other effects that are not yet well understood. Specifically, it is possible that when children assist spontaneously, their behavior is underlain by a different motivation than when they assist following direct prompts: Spontaneous prosocial behavior may reflect a genuine motivation to benefit the other, whereas cued or prompted prosocial action may be due to external pressure or a desire to adhere to social norms (Eisenberg et al., 2016). In support of this motivational interpretation, toddlers who shared after fewer cues were also found to share more with the other, suggesting they had greater intention to benefit the other (Pettygrove et al., 2013). Alternatively, it is possible that, at least for young children, spontaneous and prompted prosocial action may not differ in their underlying motive. When young children help only following cues, this may be due to their limited cognitive skills and thus their failure to comprehend how to offer help in the absence of explicit signals (Svetlova et al., 2010), rather than a reflection of less caring on the child’s part. In this case, the added cues serve as scaffolding, assisting the young child to better understand the situation and how to function in it. In support of this cognitive interpretation, studies found that compared to older toddlers, younger toddlers need more communicative cues in order to help (Svetlova et al., 2010; Brownell et al., 2013a). Moreover, better theory of mind abilities predicted young children’s spontaneous sharing, even after controlling for age (Wu and Su, 2014).

In the present study, we tried to shed light on the meaning of early spontaneous prosocial behavior, by examining its consistency—within the same task and over time. If spontaneous prosocial action is associated with greater amounts of assistance at the task, this would suggest that spontaneous responses likely reflect a stronger motivation to assist the other compared to prompted prosocial behavior. If instead (or in addition), cued or requested prosocial behavior in toddlerhood predicts spontaneous prosocial action in early childhood, then the cognitive interpretation of early spontaneous vs. prompted prosocial behavior would be supported. In addition, we explored whether spontaneity of prosocial action is consistent across subtypes and over time, questions not yet addressed by prior work.

### 1.4. The present study

The current study examined children’s prosocial behaviors longitudinally, at ages 18 and 36 months. At each age, behavioral tasks examining instrumental helping, compassionate helping, and sharing were administered. The study focused on three main research questions. First, we asked whether different types of prosocial behavior converge in toddlerhood (18 months) and early childhood (36 months). In line with some previous findings, we expected partial convergence, such that different subtypes of prosocial behavior would be modestly intercorrelated at each age (Sommerville et al., 2013; Newton et al., 2016; Schachner et al., 2018). Such finding would rule out the possibility that early prosocial behavior is purely situationally-determined.

Second, we examined the consistency over time of these different subtypes of prosocial behavior. Based on prior work (see above), we expected modest continuity in prosocial behavior from toddlerhood to early childhood. However, we did not make a specific prediction regarding the pattern of longitudinal associations within vs. across subtypes, given the paucity of prior work on this issue. As noted above, if consistency over time is shown to be substantial within each form of prosociality yet weak between different forms, this would suggest that early prosociality is domain-specific, with different prosociality subtypes developing independently and drawing on distinct mechanisms. In contrast, a pattern of similar associations within and across subtypes would suggest that different forms of prosociality are manifesting, at least to some extent, common underlying disposition and mechanisms (Kärtner et al., 2014; Knafo-Noam et al., 2015, 2018; Paulus et al., 2015; Schachner et al., 2018).

Third, we examined consistency and change in spontaneous vs. prompted prosocial behavior. To this end, in several tasks at each age, children had the opportunity to assist either spontaneously or following cues, as well as to assist a little vs. a lot. We examined whether children who act spontaneously also assist more at the task, predicting a positive association between these two aspects (Pettygrove et al., 2013); such associations would suggest that even at a young age, spontaneous prosocial behavior may signal a stronger other-oriented motivation than cued prosocial action. We further examined whether the tendency to assist spontaneously converges across tasks, both concurrently and across age. To our knowledge, previous studies did not examine the consistency of early spontaneous prosocial behavior; therefore, we did not have a specific hypothesis regarding these associations.

## 2. Methods

### 2.1. Participants

The sample consisted of 151 Israeli children (51% girls) assessed in their homes at two time-points; 138 children participated at both ages (at 18 months: *N* = 147, *M*_age_ = 18.37 months, *SD* = 0.58; at 36 months: *N* = 142, *M*_age_ = 36.95 months, *SD* = 0.85). This research is part of a larger longitudinal study following a community sample across the first 3 years of life (Davidov et al., 2021; Paz et al., 2022). No *a priori* power analysis was conducted to determine the sample size for the specific research questions of the current paper; however, prior studies that examined similar questions typically had smaller samples (Kärtner et al., 2014; Schachner et al., 2018).

Families were recruited through a major hospital in Jerusalem. A month after giving birth mothers received a letter about the study, and a month later they were recruited to the study by phone. Ethics approvals were obtained from Hadassah Medical Center, Israel’s ministry of health, and The Hebrew University’s IRB.

Families were all Jewish. The sample was predominantly of middle to low-middle SES. The median monthly family income reported by the parents (8,500–12,500 NIS) was lower than the average family monthly income in Israel at the time (18,671 NIS per month; Israel Central Bureau of Statistics, 2017). For 27% of the families, monthly income was below the 30th income percentiles, 29% were in the 30th–40th percentile range, 35% were between the 40th–70th, and 10% reported incomes above the 70th percentile. The sample was relatively educated, with 76% of mothers having a university degree. There was considerable variability in religiosity, with 29% of the mothers identifying as secular, 20% as traditional, 34% as religious, and 17% as ultra-orthodox. The number of children per family ranged from a single child to nine children (*M* = 2.84, *SD* = 1.77).

### 2.2. Procedure

Assessment was carried out at children’s homes by trained female experimenters. Only those procedures and measures relevant to the current report are detailed below. At each age, five prosociality tasks were administered: two instrumental helping tasks (out-of-reach object, finding a lost object); two compassionate helping tasks toward another in distress (distress simulations of mother and experimenter); and one task of sharing a limited resource (snack) with a sad experimenter. In five of the 10 tasks (18 months: sharing and instrumental out-of-reach; 36 months: sharing, instrumental out-of-reach, and compassionate helping to the sad experimenter) children had an opportunity to assist spontaneously, before any cue regarding how to do so was given, or to assist following cues; in the remaining five tasks, no cues were given as to how to assist (see below).

Because of young children’s limited patience, the emotional nature of some tasks, and the research questions (which focused on the links between tasks, rather than on mean-level comparisons), the order of tasks at each visit was fixed and not counterbalanced. The order of the tasks was determined in an attempt to maximize children’s completion of as many tasks as possible (for example, by interspersing the more stressful, distress-related tasks with other, neutral-affect tasks), and keeping the setting as ecologically valid as possible (for example, by performing the lost toy instrumental task immediately after that toy had been used in a preceding activity; see Supplementary material online for the order of the tasks). Children’s responses to all the tasks were videotaped for later coding. At the end of each home visit, the family received a gift card of 50 NIS (approximately $15) and a toy for the child.

Each task at each age was coded by a main coder (out of a team of graduate and undergraduate research assistants). For each task at each age, another coder independently rated a subset of 20% of the videos, randomly selected, for calculation of inter-rater reliability. In case of discrepancies, the rating of the main coder was always used.

### 2.3. Measures

#### 2.3.1. Instrumental helping

At each age, two tasks were administered by the experimenter—one task of helping to return an out-of-reach object and one task of searching for a lost object. All tasks were performed using neutral vocalization and demeanor, without expressing any distress or urgency.

##### 2.3.1.1. Out of reach pen—18 months

In this task, based on Warneken and Tomasello (2006), the experimenter pretended to unintentionally drop her pen in the child’s direction. The simulation lasted 30 s, and no eye contact was made with the child throughout. For the first 15 s, the experimenter looked at the pen and uttered “my pen” a few times in a neutral tone of voice. Then for the last 15 s, she reached out and tried to grab the pen, expressing effort to reach it but without any demonstration of distress. If the child brought the pen to the experimenter, the simulation ended. Children’s help (bringing the pen to the experimenter) was coded on a dichotomous scale, with 0 = *did not help*, 1 = *helped*. Inter-rater reliability (based on 20% of the videos) was kappa = 1.00.

Upon careful inspection, we noticed that in some of the cases the experimenters started reaching for the pen right away (instead of first just looking and exclaiming “my pen,” without reaching, as was intended); these children (*n* = 61) did not differ from the children for whom the two-stage procedure was implemented properly (*n* = 86) in the probability of helping to pick up the pen, with 53% helping in the former group and 52% helping in the latter, χ^2^(1) = 0.16, *p* = 0.90. We therefore used the helping score of the entire sample in the analysis. However, spontaneous helping (that is, before the experimenter started reaching) could only be coded for the latter children. The spontaneous helping score included 3 levels, with 0 = did not help, 1 = helped, but not spontaneously (only after the experimenter reached), 2 = helped spontaneously (before the experimenter reached for the pen). Inter-rater reliability (based on 20% of the videos) was ICC = 0.99.

##### 2.3.1.2. Out of reach crayons—36 months

In this task the experimenter pretended to accidentally drop a box of crayons. The experimenter waited for 5 s looking at the scattered crayons, then started to collect them slowly for 30 s, without making eye contact with the child. If the child helped the experimenter, she thanked the child briefly and continued collecting the crayons until the time was over. The time window for spontaneous helping was shorter in this task than in the pen task (5 vs. 15 s), because we were concerned that waiting 15 s at this age would appear artificial to children (and waiting 5 s at 18 months was too brief for children to respond).

Children’s helping attempts were coded from videos using coding software (INTERACT© by Mangold). Several scores were derived: (a) a 3-point helping score, with 0 = *did not help*, 1 = *helped a little*, and 3 = *helped a lot*. (b) spontaneous helping—whether the child began helping before the experimenter started collecting the crayons, with 0 = *did not help*, 1 = *helped but not spontaneously*, 2 = *spontaneous helping*. (c) duration of helping—a continuous score reflecting the proportion of seconds the child helped out of the total duration of the task. (d) number of crayons collected, on a 0–3 scale, with 0 = *none*, 1 = *one crayon*, 2 = *some* (2–3 crayons), 3 = *many* (four crayons or more). Inter-rater reliabilities (based on 20% of the videos) were high, Intraclass correlation (ICC) ranging from 0.92 to 0.95. Similar tasks have demonstrated validity in prior work (e.g., Bryan et al., 2014).

##### 2.3.1.3. Searching lost ball—18 months

This task was an adaption of other searching tasks used in prior work for testing instrumental helping (e.g., Liszkowski et al., 2006). During the home visit, the experimenter collected all the toys that had been used in a previous activity back into her bag and pretended she could not find a ball, which was clearly visible to the child (we verified beforehand with the mother that the child knew what the word “ball” means). The experimenter pretended to search for the ball for 30 s while saying out loud: “where is my ball?,” “I need to find it,” in a neutral tone of voice, without making direct eye contact with the child and without expressing any distress. If the child brought the ball to the experimenter or put it in her bag, the simulation was over. Children’s helping attempts were rated from the videos on a dichotomous scale, with 0 = *did not help,* and 1 = *helped* (if the child either brought the ball to the experimenter, put it in her bag, looked for the ball intensely without finding it, or pointed at the ball in an attempt to draw the experimenter’s attention to it). Inter-rater reliability (based on 20% of the videos) was kappa =1.00.

##### 2.3.1.4. Searching lost keys—36 months

This task was also adapted from previous searching tasks (e.g., Liszkowski et al., 2006). When the child was sitting across from her, ready to play a game, the experimenter put down her keys next to her, stating out loud that she is putting them there so she would not lose them. While playing the game with the child the experimenter “accidentally” placed a sheet of paper over the keys. At the end of the game (approximately 10 min after putting down the keys), the experimenter wondered where her keys were, pretending she forgot where she had put them. Then she looked for the keys in her belongings for 30 s before finding them. If the child found the keys and brought them to the experimenter, the simulation was over; likewise, if the child repeatedly pointed at the keys, the experimenter found them and the simulation was over. Helping was coded on a 3-point scale, with 0 = *did not help,* 1 = *helped a little* (the child made mild effort to help the experimenter), 2 = *helped a lot* (the child brought the keys to the experimenter, showed her where they were, or helped her look for them intensely without finding them). Inter-rater reliability (based on 20% of the sample) was ICC = 0.94.

##### 2.3.1.5. Data reduction for instrumental helping

At each age, a composite total score of instrumental helping was created as a 3 levels scale, with 0 = did not help in either task, 1 = helped in one of the tasks, and 2 = helped in both tasks.

#### 2.3.2. Compassionate helping

At each age, two distress simulations were performed, one by the experimenter and one by the mother. At 18 months, both simulations portrayed the mother/experimenter getting hurt and crying. At 36 month, the mother repeated a shorter version of the same pain simulation, whereas the experimenter performed another simulation portraying sadness, as described below.

##### 2.3.2.1. Pain simulation—18 and 36 months

The experimenter pretended to bump her knee while sitting in front of the child, and the mother pretended to hurt her finger while playing with a pounding toy. Upon getting “hurt,” the victim cried for 60 s when children were 18 months old (medium intensity cries for 30 s, and then subsiding for another 30 s). When the children were 36 months-old, a shorter version of the simulation was used, which was more appropriate at this age (the full length simulation felt too intense for the older children); the mother therefore cried for 40 s (20 s at medium intensity and then subsiding for another 20 s). At the end of the simulations, the victim made eye contact with the child, smiled, and assured the child that she was now alright. Attempts to help and comfort the distressed experimenter/mother included physically comforting her (e.g., patting, kissing, calming words; but not seeking comfort *from* the mother), trying to recruit help on her behalf (e.g., from another adult), bringing an object to her, and so on. As helping frequency at 18-months was low (see below), compassionate prosocial behavior was scored dichotomously, with 0 = *not shown*, and 1 = *shown by the child*. Inter-rater reliabilities, based on 20% of the videos coded by a second, independent rater were high, with kappa values ranging from 0.85 to 0.94.

##### 2.3.2.2. Sadness simulation—36 months

The experimenter did not perform the pain simulation at this age, but rather a sadness simulation (we thought that children at this age might be suspicious if two similar pain simulations were presented to them). The experimenter told the child excitedly that she brought her favorite doll (unisex doll of a cartoon figure) but then “discovered” that the doll’s arm had been broken. She feigned sadness for 50 s, without making eye contact, alternating between holding the doll (first 30 s), trying to fix it, and placing it between her and the child (remaining 20 s). Finally, the experimenter succeeded in fixing the doll and was happy. If the child was able to fix the doll at any point, the simulation ended. Similar simulations have been used to measure young children’s empathy and prosociality (e.g., Dunfield and Kuhlmeier, 2013). Prosocial behavior in this task was coded dichotomously (0 = *not shown*, 1 = *shown*), as well as on a 4-point scale reflecting the extent of assistance shown by the child: 0 = *none*, 1 = *brief* (a single or weak attempt), 2 = *moderate* (child tried to help/comfort a few times, or made a single intense or complex attempt), 3 = *prolonged* (child repeatedly and substantially engaged in prosociality). A 3-point spontaneity score was also coded, reflecting whether the child tried to fix the doll spontaneously, that is, even before the experimenter demonstrated how it might be repaired by trying to fix it herself, with 0 = *no prosocial behavior*, 1 = *acted prosocially, but not spontaneously*, 2 = *spontaneous prosocial action*. Inter-rater reliabilities, based on 20% of the sample, ranged from ICC = 0.97 to 1.00 for all the codes.

##### 2.3.2.3. Data reduction for compassionate helping

At each age, a composite total score of compassionate helping was created as a 3 levels scale, with 0 = not shown in either task, 1 = shown in one of the tasks, and 2 = shown in both tasks.

#### 2.3.3. Sharing

The same sharing task was performed at both ages by the experimenter. However, slight changes were made in the cues presented to the child, in order to accommodate children’s developmental level, as was done in similar simulations measuring costly sharing in prior work (Dunfield et al., 2011; Dunfield and Kuhlmeier, 2013).

##### 2.3.3.1. Sharing a snack—18 and 36 months

The experimenter told the child that she brought a snack for both of them (we verified with the mothers that the child liked this snack). While pouring the snacks into two bowls, the experimenter “discovered” that her bag of snack was empty. Handing over the full bowl to the child, she looked at her empty bowl and simulated distress. The 60 s simulation was built gradually with three progressive stages, in order to give the children more opportunities to understand how to assist the experimenter (Svetlova et al., 2010). At each age, these stages included: First, an un-cued phase, to enable spontaneous sharing. This consisted of 30 s during which the experimenter pretended to be sad for having no snack, while avoiding eye contact with the child (looking at her empty bowl) and, at 18 months only also additional 15 s in which the experimenter initiated eye contact with the toddler, shifting her gaze between the infant and the bowls (this behavior was very implicit for 18 month-olds, in contrast to the older children). The second phase was an explicit but non-verbal cue to share. At 18 months this consisted of the experimenter extending her hand toward the child while holding her bowl, still looking sad and alternating her gaze between the bowls and the child for 15 s; this cue was considered too strong for 36 month-olds, and therefore at this age the explicit cue consisted of the experimenter making eye contact with the child and altering her gaze between the child and the two bowls while looking sad, for 20 s. The final stage at both ages was a direct verbal request: the experimenter asked the child directly, only once, if the child would like to give her some of his/her snack. If the child shared any amount at any stage, the simulation ended.

Two codes were used: (a) Stage of sharing, a 4-level scale with 0 = *did not share*, 1 = *compliance* (shared after direct verbal request), 2 = *cued sharing* (shared when experimenter’s hand was extended at 18 months, or when eye contact was made at 36 months), 3 = *spontaneous sharing*. (shared before the latter cues, noted for a code of 2, were given) (b) The amount of snack shared by the child, a 4-level scale with 0 = *did not share*, 1 = shared *one piece*, 2 = shared *some* (a handful), 3 = shared *most of his/her snack*. Inter-rater reliabilities (ICCs, based on 20% of the videos) ranged from 0.93 to 0.99. Thirteen episodes at 18 months could not be coded, due to experimenter error or parental interference.

#### 2.3.4. Control variables

Mothers completed a demographic questionnaire at each home visit, and items from it were examined as potential control variables (e.g., maternal age in years, years of maternal education, a 7-point family income item). Child temperament was reported by mothers at 12-months using the short form of the Infant Behavior Questionnaire-Revised (Putnam et al., 2014). This questionnaire includes 91 items, rated on 1–7 scales, assessing 14 aspects of temperament, which comprise three broad temperamental dimensions: Negative Emotionality, Positive Affectivity/Surgency, and Orienting/Regulatory Capacity. These three broad dimensions (average scores) were examined as potential control variables in the present study (for the psychometric properties of this instrument, see Putnam et al., 2014).

## 3. Results

### 3.1. Descriptive statistics and preliminary analyses

Descriptive information is presented in Table 1 for the instrumental and compassionate helping tasks and in Table 2 for the sharing task. As can be seen, at both 18 and 36 months, the majority of children helped instrumentally at least once (72% and 76%, respectively; about a quarter of the children at each age helped in both instrumental tasks). Wilcoxon Test, a non-parametric test for paired samples, comparing the rates of instrumental helping (total score) at 18 and 36 months, showed no significant change with age, *Z* = −0.387, *p* = 0.699. In contrast, the majority of 18 month-olds did not show compassionate helping toward either the distressed experimenter or mother (with only 41% acting prosocially in at least one task, more typically toward the distressed mother). Similarly, the majority of toddlers did not share their snack with the distressed experimenter (with only 37% sharing at any stage of the task at 18 months; see Table 2). These two prosocial behavior subtypes were more prevalent by 36 months, with 65% of the children showing compassionate helping in at least one task (approximately equally toward the mother and the experimenter), and 72% of the children sharing with the experimenter, at any stage of the task, at this age. Wilcoxon Tests showed that the increase with age was significant for both compassionate helping, *Z* = 4.77, *p* < 0.001, and sharing, *Z* = 4.73, *p* < 0.001.

**Table 1:** Descriptive statistics for instrumental and compassionate helping tasks.

Measure^a^	Prevalence (% of children who assisted at all)	M (SD)^b^	% who assisted spontaneously^c^
**Instrumental helping**			
Pen 18 m (0–1)	52.5%		84.44%^d^
Bal 18 m (0–1)	52.5%		
Crayons 36 m (0–1)	59.2%		40.47%
Keys 36 m (0–2)	45.1%	0.83 (0.90)	
Instrumental-total 18 m (0–2)	71.9%	1.06 (0.79)	
Instrumental-total 36 m (0–2)	76.1%	1.04 (0.72)	
**Compassionate helping (comforting)**			
Pain mother 18 m (0–1)	37.1%		
Pain experimenter 18 m (0–1)	10.2%		
Pain mother 36 m (0–1)	40.3%		
Sad experimenter 36 m (0–3)	46.5%	1.08 (1.29)	61.66%
Compassionate-total 18 m (0–2)	40.8%	0.46 (0.60)	
Compassionate-total 36 m (0–2)	64.8%	0.85 (0.73)	

a The rating scale of each prosocial measure is noted in brackets following the variable name.

b Means and SDs for dichotomous measures are not shown (because they are redundant with the %s reported in the first column).

c Calculated out of those children who assisted at the task (to any extent), in tasks where both spontaneous and cued assistance were possible.

d The rate shown is for spontaneous helping within 15 s; for spontaneous helping within the first 5 s, the rate was 35.55% of the helpers.

**Table 2:** Descriptive statistics for sharing task.

	18 months	36 months
Prevalence (% who shared at all)	36.9%	72.1%
M (SD) stage of sharing^a^	0.61 (0.89)	1.14 (0.99)
M (SD) amount shared^a^	0.64 (0.95)	1.40 (1.05)
**Out of those who shared, % of children sharing…**		
Spontaneously	10.4%	20.8%
After a non-verbal cue (cued)	43.8%	15.8%
After a verbal request (compliance)	45.8%	63.4%

a 0–3 rating scale.

The rates of spontaneous prosocial behavior are also included in Tables 1, 2. For instrumental helping, 85% of the toddlers who helped in the pen task at 18 months did so spontaneously, within 15 s (when only the first 5 s were examined, 35.6% of the helpers did so spontaneously). At 36 months, 40% of the children who helped pick up the scattered crayons did so spontaneously (i.e., in the first 5 s, before any cue was given). For sharing behavior, spontaneous sharing of the snack was rare at 18 months, with only 10% of the sharers doing so, compared to 21% of sharers at 36 months. For compassionate helping, only one task at 36 months enabled both spontaneous and cued assistance, and 61% of the children tried to help the sad experimenter fix her doll before any cue regarding how to do so was given.

Additional analyses showed that none of the prosocial behavior scores, for any of the tasks at either age, was significantly associated with the demographic variables, including: maternal age, all *r*s between −0.11 and 0.12, *p >* 0.192, maternal education, *r*s between −0.09 and 0.13, *p* > 0.127, and family income, *r*s between −0.12 and 0.14, *p > 0*.126. Likewise, child’s gender was not significantly associated with the prosociality scores, albeit two gender difference approached significance (for Ball 18-months *t* = −1.93, *df* = 139, *p* = 0.055, *M*_male_ = 0.44 (0.50) *M*_female_ = 0.60 (0.49); for Pen 18-months *t* = 1.78, *df* = 139, *p* = 0.077, *M*_male_ = 0.60 (0.49) *M*_female_ = 0.45 (0.50); for all others measures, *t*s ranged from 0.014 to 1.36, all *p* > 0.178). Of the three temperament measures, Positive Affectivity/Surgency at 12-months was correlated with several prosocial scores: total instrumental helping at 18 months, total compassionate helping at 36 months, and with both amount and stage of sharing at 36 months, all *r*s between 0.20–0.25, *ps* from 0.021 to 0.003. No other associations with temperament were found. Only Positive Affectivity/Surgency was therefore included as a control variable.

### 3.2. Consistency of individual differences across prosocial subtypes and age

#### 3.2.1. Overview of analysis

To examine our main research questions regarding convergence across prosociality subtypes and continuity over time, we used two sets of analyses. First, for a simple examination of consistency, we computed the correlations between the prosociality measures, using the total instrumental and compassionate helping scores and the two sharing scores (stage and amount) at each age. Both zero-order correlations and partial correlations controlling for Positive Affectivity/Surgency, were examined. However, correlations between observed variables can be substantially affected by differences in measurement error between the various scores. To mitigate this problem, a second set of analyses used Structural Equation Modeling (SEM), in which latent variables of the prosocial subtypes were estimated at each age from the observed task scores, and the concurrent and longitudinal associations among the prosocial subtypes were then examined among the latent variables (which partial out measurement error; Stephenson and Lance Holbert, 2003; Coffman and MacCallum, 2005). The Analysis was conducted using the lavaan package in R (Rosseel, 2012). Full information maximum likelihood (FIML) was used for treating missing data with Maximum likelihood estimator (ML). All observed scores were standardized prior to being entered into the models.

As a preliminary step to the SEM analysis, we examined separate measurement models at each age. First, a single factor model was examined, in which all six observed prosocial scores loaded on a single prosociality factor. Support for this model can indicate that the different subtypes of prosociality all reflect the same general, global prosocial disposition. The single factor model was then compared to a model containing three latent prosocial variables corresponding to the three subtypes—instrumental, compassionate, sharing—each estimated from two observed scores. For instrumental and compassionate, the two relevant tasks at each age were used as the observed indicators, and for sharing at both ages, stage of sharing and the amount of snack shared were used as the two observed indicators. The covariances between the latent factors were also estimated at each age, to examine the concurrent links between subtypes of prosociality. Better fit for the 3-factor model compared to the 1-factor model would support the interpretation that the three subtypes of prosociality are distinct and likely underlain by different mechanisms. Finally, we conducted the main SEM analysis, modeling the regression pathways between all latent factors at 18-months and all latent factors at 36 months (in addition to the concurrent covariances), in order to examine the longitudinal associations both within and across the subtypes of prosociality.

#### 3.2.2. Links between observed prosocial behavior scores (correlations)

Table 3 presents the correlations between the different forms of prosociality within age (Pearson correlations are presented; Spearman correlations were highly similar, see Supplementary Table S1). Consistent with hypothesis, the findings show partial convergence between different subtypes of prosociality within age, at both 18 and 36 months. At 18 months, toddlers who offered compassionate helping also helped more in the instrumental tasks and shared more from their snack. Instrumental helpers also tended to share their snack at an earlier stage. Similarly, at 36 months, compassionate helping was linked with greater instrumental helping as well as with sharing of larger amounts and at an earlier stage. Partial correlations, controlling for the temperament dimension of Positive Affectivity/Surgency, were virtually identical (see Supplementary Table S2).

**Table 3:** Correlations between different subtypes of prosociality at each age.

	Instrumental total	Compassionate total	Sharing stage	Sharing amount
Instrumental total	−	0.34^**^	0.18^*^	0.11
Compassionate total	0.27^**^	−	0.13	0.19^*^
Sharing stage	0.10	0.26^**^	−	0.83^**^
Sharing amount	0.12	0.24^**^	0.74^**^	−

† *p* ≤ 0.10.

* *p* < 0.05.

** *p* < 0.01 (all two-tailed).

Pearson correlations are presented. Correlations at 18 months are presented above the diagonal, and correlations at 36 months are presented below the diagonal.

Table 4 presents the longitudinal associations between the different forms of prosociality. As shown, modest associations emerged both within and across subtypes. Correlations within each subtype, located on the diagonal, emerged for all subtypes (marginally for instrumental helping). Correlations across subtypes, located off the diagonal, were also found, and were similar in magnitude to the links within subtypes. Thus, instrumental and compassionate helping at 18 months were both significantly associated with sharing amount at 36 months, and sharing amount and stage at 18 months were marginally associated with instrumental helping at 36 months. The pattern of Spearman correlations was very similar (see Supplementary Table S3), as were the partial correlations, controlling for children’s Positive Affectivity/Surgency (see Supplementary Table S4).

**Table 4:** Longitudinal links from 18 to 36 months, between different subtypes of prosocial behavior.

	18 months			
	Instrumental total	Compassionate total	Sharing stage	Sharing amount
**36 months**				
Instrumental total	0.15^†^	0.00	0.16^†^	0.17^†^
Compassionate total	0.07	0.24^**^	−0.05	−0.05
Sharing stage	0.12	0.07	0.22^*^	0.15^†^
Sharing amount	0.19^*^	0.23^**^	0.24^**^	0.23^*^

† *p* ≤ 0.10.

* *p* < 0.05.

** *p* < 0.01 (all two-tailed).

Pearson correlations are presented.

#### 3.2.3. Links between latent prosocial behavior variables (SEM)

The single factor models showed poor fit to the data at both 18 months, χ^2^ = 32.08, *df* = 9, *p* < 0.001, CFI = 0.87, TLI = 0.79, RMSEA = 0.132, SRMR = 0.087, and 36 months, χ^2^ = 20.21, *df* = 9, *p* < 0.001, CFI = 0.92, TLI = 0.86, RMSEA = 0.094, SRMR = 0.065. In comparison, the 3-factor measurement model fit the data well at both 18 months, χ^2^ = 8.80, *df* = 6, *p* = 0.185, CFI = 0.98, TLI = 0.96, RMSEA = 0.056, SRMR = 0.061, and 36 months, χ^2^ = 7.08, *df* = 6, *p* = 0.313, CFI = 0.99, TLI = 0.98, RMSEA = 0.036, SRMR = 0.039. The fit for the 3-factor model was significantly better than that for the single factor model at both 18 months, χ^2^ difference test = 23.28, *df* = 3, *p* < 0.001, and 36 months, χ^2^ difference test = 13.12, *df* = 3, *p* = 0.004. However, at both ages, the covariance matrix of the latent factors was not positive-definite, suggesting a different structure may suit the data better. Specifically, high covariances between two of the latent variables, instrumental and compassionate, indicated possible linear dependency or redundancy between them, which may suggest they are not both needed for capturing the structure of the data (Wothke, 1993). Further examination indeed revealed that a 2-latent factor model was most appropriate for the data at both ages. In this model, instrumental and compassionate helping were combined into one latent factor, “instru-compassionate,” which was estimated from all four observed instrumental and compassionate scores; the second latent factor was sharing, which was estimated from the two observed scores of the sharing task (stage and amount). This model had a positive-definite covariance matrix, and showed excellent fit to the data at both 18 months, χ^2^ = 10.27, *df* = 8, *p* = 0.256, CFI = 0.99, TLI = 0.98, RMSEA = 0.043, SRMR = 0.035, and 36 months, χ^2^ = 5.20, *df* = 7, *p* = 0.635, CFI = 1.00, TLI = 1.03, RMSEA < 0.001, SRMR = 0.038 (at 36 months, a covariance between two observed scores, sad experimenter and sharing stage, was also added to the measurement model, to prevent a negative estimation of the variance of sharing stage). The covariance between the two latent factors (instru-compassionate and sharing) was significant at both 18 months, β = 0.30, *p* = 0.002, and 36 months, β = 0.34, *p* = 0.014.

The 2-factor model fit the data better than the single factor model at both 18 months, χ^2^ difference test = 21.96, *df* = 1, *p* < 0.001, and 36 months, χ^2^ difference test = 15.00, *df* = 2, *p* < 0.001, and its fit was not significantly different from that of the three-factor model at both ages, χ^2^ difference tests < 1.32, *p* > 0.51. Therefore, the 2-factor measurement models, which reflects both convergence and differentiation between subtypes of prosociality, were used in the longitudinal SEM.

**Figure 1:**
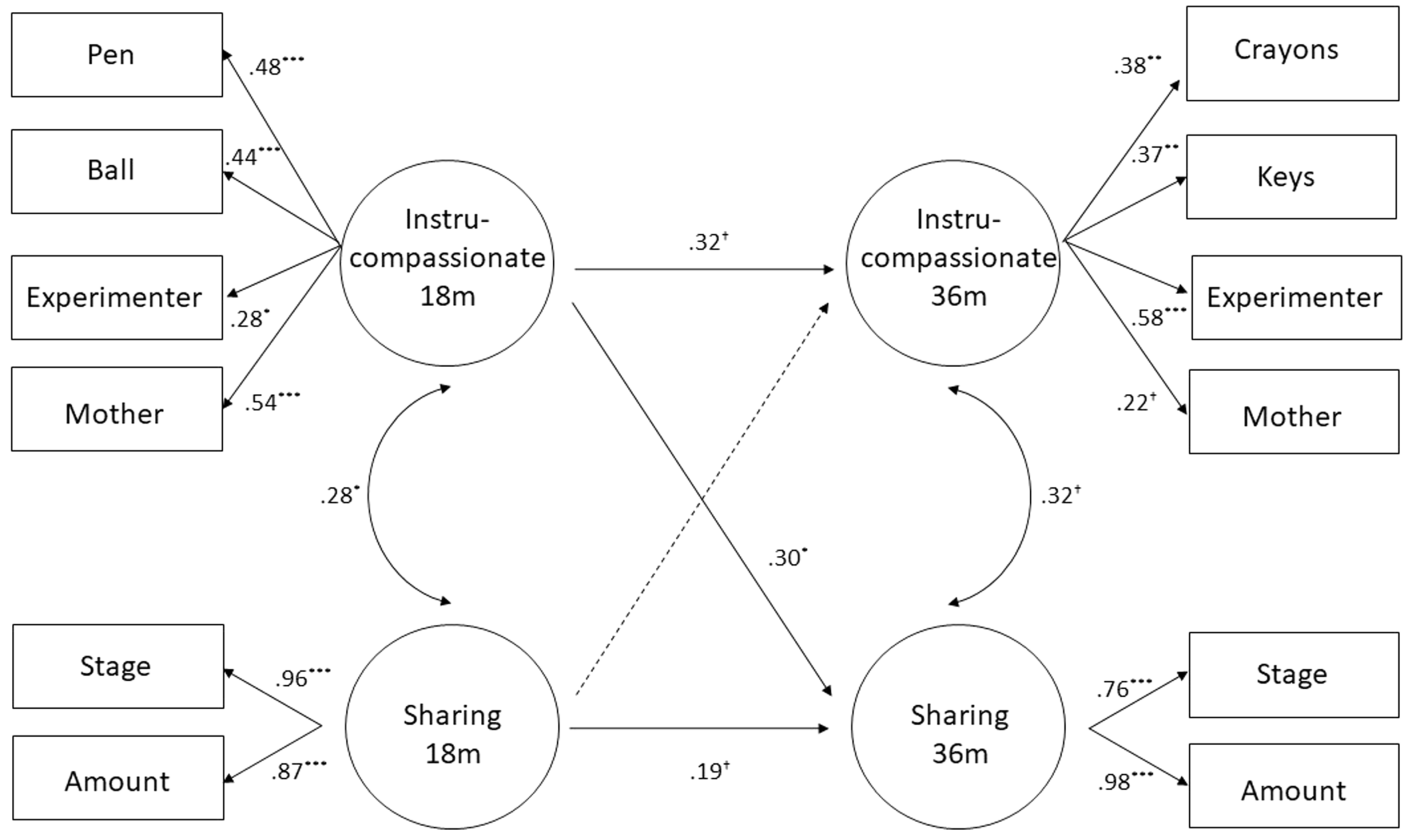
SEM model examining concurrent and longitudinal associations between latent variables of the two factors prosociality subtypes at 18 and 36 months. Significant paths and covariances appear in solid arrows, with standardized coefficients, and non-significant ones are in dotted arrows. ^†^*p* < 0.10; ^*^p < 0.05; ^**^p < 0.01; ^***^p < 0.001 (all two-tailed).

For the SEM, we modeled four longitudinal regression paths between the 18 months and 36 months latent factors. The model had reasonable fit χ^2^ = 72.03, *df* = 48, *p* = 0.014, CFI = 0.93, TLI = 0.91, RMSEA = 0.058, SRMR = 0.062. The model is presented graphically in Figure 1 and standardized parameter estimates are reported in Table 5. As can be seen, three of the four regression paths were significant or close to significance. Within subtypes, the longitudinal pathways from 18 to 36 months approached significance for both instru-compassionate and sharing. Across subtypes, instru-compassionate helping at 18 months predicted sharing at 36 months (but early sharing did not predict subsequent instru-compassionate helping).

**Table 5:** Standardized parameter estimates from final SEM model.

Latent variable	Observed indicators	Estimate	*p*
Instru-compassionate 18 m	Pen	0.48	<0.001^***^
	Ball	0.44	<0.001^***^
	Distressed experimenter	0.28	0.016^*^
	Distressed mother	0.54	<0.001^***^
Sharing 18 m	Stage	0.96	<0.001^***^
	Amount	0.87	<0.001^***^
Instru-compassionate 36 m	Crayons	0.38	0.002^**^
	Keys	0.37	0.004^**^
	Distressed experimenter	0.58	<0.001^***^
	Distressed mother	0.22	0.078^†^
Sharing 36 m	Stage	0.81	<0.001^***^
	Amount	0.92	<0.001^***^
**Longitudinal regressions**			
Instru-compassionate 36 m	Instrumental 18 m	0.32	0.096^†^
	Sharing 18 m	0.05	0.761
Sharing 36 m	Instrumental 18 m	0.30	0.030^*^
	Sharing 18 m	0.19	0.078^†^
**Concurrent covariances**			
Instru-compassionate 18 m	Sharing 18 m	0.28	0.038^*^
Instru-compassionate 36 m	Sharing 36 m	0.32	0.074^†^

Model fit indexes: χ^2^ = 72.03, df = 48, *p* = 0.014, CFI = 0.93, TLI = 0.91, RMSEA = 0. 058, SRMR = 0.062.

† *p* < 0.10.

* *p* < 0.05.

** *p* < 0.01.

*** *p* < 0.001 (all two-tailed).

### 3.3. Spontaneous vs. cued prosocial behavior: Its consistency and meaning

To examine the role of spontaneous prosocial behavior, we took three steps. First, we examined whether the tendency to assist spontaneously was consistent across different tasks, within each age (two tasks at 18 months, three tasks at 36 months). Second, we examined whether the tendency to assist spontaneously was consistent across age. Finally, we addressed the links between spontaneity and degree of prosocial behavior, by examining whether children who assisted spontaneously also tended to assist more in the task (e.g., helped for a longer duration, or shared greater amounts) than children who assisted only following prompts.

At 18 months, there was no association between spontaneous helping in the instrumental task (pen) and spontaneous sharing of the snack, χ^2^(6) = 4.51, *p*  = 0.608 (the results did not change when spontaneous helping in the pen task was examined within the first 5 s, instead of 15 s). At 36 months, two of three potential associations were significant: Children who helped spontaneously in the instrumental task (dropped crayons) were also more likely to do so in the compassionate helping task (sad experimenter—broken doll): χ^2^(4) = 10.93, *p* = 0.027; and children who shared their snack spontaneously were also more likely to show spontaneous compassionate helping: χ^2^(6) = 20.98, *p* = 0.002. Thus, some consistency in spontaneous helping across different prosociality tasks appeared to emerge by early childhood (36 months). Notably, for the two prosocial tasks that correspond to those used in toddlerhood (instrumental out-of-reach and sharing), the tendency to assist spontaneously was unrelated at 36 months either, χ^2^(6) = 8.83, *p* = 0.184.

As for longitudinal associations, only one significant link was found: between spontaneous sharing at 18 and 36 months (no other longitudinal links emerged for spontaneity of sharing, and none were found for spontaneity of instrumental helping in the pen task, either when the first 5 s or the first 15 s were considered as spontaneous helping, χ^2^ between 1.08 and 7.61, all *p* > 0.107). Table 6 presents the cross-tabulation of sharing stages at the two ages, χ^2^(9) = 17.19, *p* = 0.046. As shown, toddlers who shared spontaneously at 18 months were significantly more likely to also share spontaneously at 36 months, *Z* = 2.70, *p* = 0.007. Interestingly, toddlers who shared after being given a non-verbal cue at 18 months also tended to later share spontaneously at 36 months, *Z* = 1.90, *p* = 0.057. Conversely, toddlers who shared only after a direct request (compliance) were unlikely to share spontaneously by 36 months, *Z* = −2.10, *p* = 0.036 (see Table 6). In fact, there was also evidence that compliant sharers were reluctant to share: When compliant sharing and non-sharing were collapsed into one category, toddlers who showed either of these behaviors at 18 months were likely to also behave similarly at 36 months, compared to toddler who shared after a cue or spontaneously at 18 months, *Z* = 2.2, *p* = 0.028.

**Table 6:** Cross-tabulation of sharing stages at 18 and 36 months.

	36 months			
	No sharing (*n* = 33)	Compliance (*n* = 54)	Cued (*n* = 14)	Spontaneous (*n* = 20)
**18 months**				
No sharing (*n* = 78)	24 (1.2)	33 (−0.7)	10 (0.6)	11 (−1.0)
Compliance (*n* = 19)	7 (1.0)	10 (0.8)	2 (−0.2)	0 (−2.1)
Cued (*n* = 19)	2 (−1.8)	9 (0.3)	2 (−0.2)	6 (1.9)
Spontaneous (*n* = 5)	0 (−1.4)	2 (−0.2)	0 (−0.8)	3 (2.7)

χ^2^(9) = 17.19, *p* = 0.046. Cells indicate the frequency (*n*) of each combination and, in brackets, the adjusted standardized residual, which reflects the difference between the expected and observed values as a Z score; all values greater than |1.96| are significant at *p* < 0.05 or less.

Finally, in four of the tasks, children had the opportunity to show variability in the level of spontaneity as well as in the amount of assistance they provided (make more attempts to fix the doll, and pick up more crayons at 36 months; share more from their snack at 18 and 36 months). Consistent with prediction, in all these tasks spontaneous prosociality was associated with greater assistance. For sharing, the correlation between stage of sharing and amount shared (excluding non-sharers from this analysis, so as not to inflate the correlations) was significant at both 18 months, *r* = 0.31, *p* = 0.032, and 36 months *r* = 0.36, *p* < 0.001 (see also Supplementary Table S5 for the cross-tabulation of sharing stage and amount). Similarly, in the sad experimenter task at 36 months, children who helped spontaneously also received a higher mean rating of their overall attempts to help or comfort the experimenter, reflecting greater attempts and effort to help, *t*(58) = −7.27, *p* < 0.001, respective means for spontaneous and cued: 2.86 (*SD* = 0.42) and 1.78 (*SD* = 0.73). And in the out-of-reach instrumental task at 36 months, children who helped spontaneously also helped more than the children who helped only after a cue (i.e., after the experimenter started to collect the fallen objects herself): They picked up more dropped items, *t*(82) = −2.91, *p* = 0.002, respective means (on a 0–3 scale): 2.97 (*SD* = 0.17) and 2.60 (*SD* = 0.73), and they helped for a longer duration of the time (expressed as proportion of seconds the child helped out of the total duration of the task), *t*(82) = −8.01, *p* < 0.001, respective means: 0.91 (*SD* = 0.08) and 0.52 (*SD* = 0.28). Thus, in all cases, spontaneous prosocial behavior was consistent with greater amounts of assistance to the needy other.

## 4. Discussion

The current study examined the early development of three main prosociality subtypes: instrumental helping, compassionate helping (or comforting), and sharing, from toddlerhood to early childhood. Whereas prior work has typically focused on mean levels of early prosocial behaviors (e.g., Warneken and Tomasello, 2006; Svetlova et al., 2010; Dunfield et al., 2011), our focus was on patterns of individual differences in these behaviors, particularly their consistency. Using a large longitudinal sample of typically developing children, we examined three main questions: Whether children’s tendency to assist in one way is linked to their tendency to act prosocially in other ways?; Whether children’s tendency to assist at 18 months is linked to their tendency to act prosocially at 36 months, both within the same subtype of prosociality and across subtypes?; And whether children’s tendency to help others spontaneously is consistent across subtypes of prosociality and across age, as well as linked to greater degrees of assistance? Together, these three questions address fundamental issues regarding the meaning and structure of early prosocial behavior, particularly, whether it reflects a dispositional tendency, and how broad and stable this tendency appears to be.

### 4.1. Consistency of children’s prosocial responses across subtypes and age

Consistent with hypotheses and in line with prior work (Sommerville et al., 2013; Brownell et al., 2013a; Newton et al., 2016; Schachner et al., 2018), the findings indicate that different types of prosociality converge partly in both toddlerhood and early childhood. The present study also provided new information, by showing that this partial convergence across subtypes occurred not only concurrently, but also longitudinally. This was shown both at the level of the observed variables (correlations), after controlling for temperament (partial correlations), as well as at the level of latent variables (SEM), thus reducing potential biases due to differences in measurement error (Stephenson and Lance Holbert, 2003; Coffman and MacCallum, 2005) and controlling for concurrent associations in the model. At the correlational level, at each age children who assisted a needy other in one way were also more likely to assist her in other ways. Moreover, toddlers who acted prosocially in one way at 18 months were more likely to show that same prosocial behavior at 36 months, and also more likely to show other types of prosocial behavior in early childhood. The SEM results, even more than the correlations, revealed the interrelatedness of different subtypes of prosociality. Thus, at both ages, instrumental and compassionate helping measures loaded on the same latent factor. Moreover, this combined factor was significantly associated with the sharing latent factor at each age as well as longitudinally.

This evidence of consistency in children’s prosocial behavior across subtypes and age support the notion that a moderately stable disposition (or temperamental dimension) toward prosociality is already evident during early ontogeny (Knafo et al., 2008; Knafo and Israel, 2012). Thus, toddlers’ and young children’s prosocial responses are not determined solely by situational or transient factors, but are also a reflection of trait-like tendencies to assist others and further their needs (or not to do so). The extent of a child’s tendency to act prosocially is likely co-determined by genetic and socialization factors, and their interplay (Dahl and Brownell, 2019).

At the same time, there was also evidence for differentiation between the subtypes of prosociality. First, at both ages a single factor model did not fit the data well; two latent factors were needed in order to capture the structure of the data. Moreover, although instrumental helping and compassionate helping loaded on the same latent factor, they had very different patterns of frequencies and change with age. Instrumental helping was much more frequent than compassionate helping at 18 months. Furthermore, compassionate helping increased substantially from 18 to 36 months, whereas the frequency of instrumental helping did not change across this same period. Thus, instrumental and compassionate helping are distinct, yet interrelated, subtypes of prosociality.

Instrumental and compassionate helping likely loaded on the same latent factor because they share key motivational or cognitive underlying mechanisms (see below). Sharing scores, on the other hand, loaded on a different factor. Unlike instrumental and compassionate helping, sharing involved a tangible cost to the self—that is, giving up one’s own valued resources for the benefit of the experimenter; this feature may have distinguished sharing from the other forms of helping. We note that the specific factor structure of prosocial subtypes may vary across different studies as a function of children’s age and features of the methodology (Thompson and Newton, 2013; Knafo-Noam et al., 2015, 2018). For example, in the current study, the two indicators of the sharing latent factor came from the same task, rather than from separate tasks. As well, all measures used structured observations, with the experimenter serving as the target of prosocial action in most of the tasks. Altering these or other features may affect the factor solution. But more important than the specific factor structure is the overall meaning of the results—namely, that prosocial subtypes are both distinct and interrelated. This complex pattern suggests that different facets, or subtypes, of prosocial behavior have both common and unique underlying mechanisms (Knafo-Noam et al., 2015; Davidov et al., 2016).

Common mechanisms that promote multiple forms of prosociality, thereby leading to associations between them, include both motivational and cognitive factors. For example, strong other-oriented motivation, such as concern for others’ welfare or sensitivity to others’ needs, can compel children to try and assist needy others in different ways (Eisenberg et al., 2016). And social-cognitive capabilities, such as the ability to understand others’ needs and wishes (e.g., Theory of Mind, perspective taking), may also promote assistance in multiple situations (Eisenberg et al., 2006). Interestingly, however, broad temperamental dimensions, such as the tendency to show positive affectivity, did not account for the associations between prosociality subtypes in the current study.

The more specific mechanisms contributing to each subtype likely reflect unique elements inherent to specific forms of prosociality. For example, the child’s ability to regulate the negative emotional arousal induced by another’s distress should be primarily relevant in compassionate helping situations (Davidov et al., 2016), whereas an understanding of ownership is specifically relevant for sharing (Brownell et al., 2013a). Both the common and more specific cognitive, affective, regulatory, and motivational mechanisms are likely influenced by genetic factors, as well as by environmental factors, such as social interactions with caregivers (Knafo-Noam et al., 2015; Dahl and Brownell, 2019).

### 4.2. Spontaneous vs. prompted prosocial behavior

The present study was one of the first to delve into the meaning and development of spontaneous vs. cued prosocial behavior. Developmentally, the findings showed that children’s tendency to help spontaneously, before any cue regarding helping expectations or how to assist is given, is at least somewhat consistent across situations by early childhood (36 months). Moreover, spontaneity of sharing, in particular, showed consistency from 18 to 36 months. Further research, assessing additional relevant situations in toddlerhood, is needed in order to clarify whether the tendency to assist others spontaneously might already show consistency across situations at this age.

Regarding the meaning of young children’s spontaneous vs. cued sharing, some aspects of the findings provided support for a motivational interpretation, and another aspect suggested a cognitive interpretation. According to a motivational interpretation, spontaneous prosocial action reflects a stronger other-oriented motivation compared to cued prosocial behavior, indicating a stronger desire to assist the other and to see the other satisfied; the cognitive interpretation suggests that young children may not assist spontaneously not because they do not care, but because they do not yet understand how to help the other person and thus need cues to overcome the cognitive gap (Svetlova et al., 2010; Wu and Su, 2014). Although different, the two explanations are not mutually exclusive; for example, they may apply to different children or to different situations. Indeed, different features of the current findings appear to support each explanation. The finding that toddlers who at 18 months shared following a non-verbal cue tended to become spontaneous sharers at 36 months is consistent with the cognitive interpretation. Thus, it appears that at a younger age some children needed a cue in order to understand how to assist the other, whereas at a later age those same children no longer needed the cue and were thus able to share spontaneously.

At the same time, the finding that children who acted spontaneously also assisted the other more in the task appears to support the motivational explanation (Pettygrove et al., 2013). Toddlers and young children who shared spontaneously gave the experimenter more of their snack, and children who helped spontaneously dedicated more time and effort to picking up the crayons or to fixing the doll. These findings support the motivational explanation, because if the difference in helping was only due to cognitive barriers, then once children understood (from the cue) how to assist the other, they should have done so to the same extent. This is particularly true in the sharing and crayons tasks, where spontaneous action did not provide a greater opportunity to help (in the sharing task, the procedure ended once the child shared any amount, and in the crayons task the period for spontaneous action was only 5 s); in the broken doll task, the first 30 s enabled spontaneous helping and the task continued when children helped, so children who acted spontaneously had more time to provide greater assistance. However, the consistent findings across the four tasks, and similar prior findings (Pettygrove et al., 2013), indicate that the association between spontaneity and amount of assistance is not merely a confound. Taken together, then, the present findings suggest that spontaneous vs. cued prosocial behavior in young children reflects both cognitive and motivational processes.

The current study also shows that prosocial behavior (sharing) that occurs only after a direct request (i.e., compliance), is likely not motivated by other-oriented concerns. In contrast to toddlers who shared after a non-verbal cue, those who shared only after a direct request at 18 months were unlikely to share spontaneously at 36 months, and were prone to once again share only after a request or not at all at the later age. Together with the fact that they shared the smallest amounts among the children who shared, these findings indicate that compliant prosocial behavior was likely motivated not by concern for the other’s well-being, but rather by a more self-focused motive (such as a desire to escape an uncomfortable situation; Eisenberg et al., 2016). More research is needed in order to examine this possibility, not only in sharing tasks (as in the current study) but in additional subtypes of prosociality as well.

### 4.3. Limitations and future directions

The current findings should be interpreted in light of some limitations. The study included only one sharing task at each age, and thus the sharing latent factor was based on two different responses from the same task (stage and amount), rather than on two independent tasks, which may have affected the results. Moreover, the five prosocial tasks were not counterbalanced, and the mother was the target of assistance only in the assessment of compassionate helping (one task). These features of the design precluded the comparison between the different prosociality subtypes (which indeed was not our focus; we were primarily interested in the associations between the different subtypes).

Furthermore, not all tasks included an option for spontaneous vs. cued helping, and only in the sharing task children were asked directly if they wanted to help the other. As well, in the pen task, the sample for examining spontaneous helping was reduced due to experimenter errors. Thus, it is unclear whether the findings regarding spontaneous helping apply equally to all three subtypes of prosociality, and further research is needed to better understand the meaning of spontaneous prosocial actions in different situations, particularly in toddlerhood. It should also be noted that differences in measurement error could affect the pattern of associations between measures; although we tried to address this issue by examining latent variables, it would also be important to see whether the present results are replicated in future studies, using a variety of different measures.

Nevertheless, this study also has considerable strengths. It uses longitudinal data and multiple observational measures. Furthermore, it is one of the first studies to examine the associations between the three subtypes of prosociality both concurrently and longitudinally, the first to do so from toddlerhood to early childhood, and one of the only studies to address the consistency and meaning of spontaneous vs. cued prosocial action.

The findings raise interesting questions for future research. One future direction is to identify the common mechanisms that lead to the shared variance between subtypes of prosocial behavior in the early years, as well as the mechanisms contributing uniquely to specific subtypes. Motivational, emotional, cognitive, biological, regulatory, and socialization processes may all be implicated; a better understanding of their respective roles and their interplay will deepen understanding of early prosocial development. Second, it would be of interest to examine whether the associations among subtypes of prosocial behavior in the early years vary as a function of the socio-cultural context. Different cultures emphasize different forms of prosociality (Köster et al., 2016; Davidov and Grusec, in press) and this may alter the development, meaning, and consistency of different prosociality subtypes. Third, from an applied direction, it could be valuable to investigate children who consistently show little or no prosocial behavior during the early years (Paz et al., 2022). Better understanding of the factors that contribute to this tendency, of the risks that this tendency poses for children’s adaptive functioning both concurrently and longitudinally, and of the factors that augment or mitigate such risk, would be highly useful for designing effective prevention programs.

In conclusion, the present findings contribute to a better understanding of the early development of compassion and its different manifestations in young children. In particular, they show that already at 18 months, children manifest their capacity to care and their desire to enhance the welfare of others in multiple ways – ways which are distinct yet interrelated, and modestly consistent during early development.

## Data availability statement

The original contributions presented in the study are included in the article/Supplementary material, further inquiries can be directed to the corresponding author.

## Ethics statement

The studies involving human participants were reviewed and approved by Hadassah Medical Center, Israel’s Ministry of Health, and The Hebrew University’s IRB. Written informed consent to participate in this study was provided by the participants’ legal guardian/next of kin.

## Author contributions

MD, RR-H, and CZ-W developed the overall study concept. YP, MD, TO, MH, RR-H, and CZ-W contributed to the study design. Data collection was coordinated by YP and TO who also performed a significant portion of the experiments and training of the other experimenters. The coding scheme was adapted by MD, YP and YP, MH, and TO coded the children’s responses. YP performed the data analysis and drafted the manuscript, with the aid and supervision of MD and critical comments from TO, MH, RR-H, and CZ-W. All authors contributed to the article and approved the submitted version.

## Funding

This research was supported by a US-Israel Binational Science Foundation Grant (No. 2011101) to MD, CZ-W, and RR-H. YP was supported by the Ariane de Rothschild Women Doctoral Fellowship.

## Conflict of interest

The authors declare that the research was conducted in the absence of any commercial or financial relationships that could be construed as a potential conflict of interest.

## Publisher’s note

All claims expressed in this article are solely those of the authors and do not necessarily represent those of their affiliated organizations, or those of the publisher, the editors and the reviewers. Any product that may be evaluated in this article, or claim that may be made by its manufacturer, is not guaranteed or endorsed by the publisher.
