# Supplementary material for: Prosocial behavior in toddlerhood and early childhood: Consistency across subtypes and over time
Source: Front Psychol. 2023 Feb 23;14:950160. doi: 10.3389/fpsyg.2023.950160 (PMC9997644; doi:10.3389/fpsyg.2023.950160)
Supplement: Supplementary file 1 [file Data_Sheet_1.docx]

**Prosocial Behavior in Toddlerhood and Early Childhood: Consistency Across Subtypes and Over Time**

**Supplementary Material**

**Order of tasks in home visits – 18 months**

1. Consent form

2. Additional task ^a^

3. Pain simulation (1) – mother or experimenter ^b^  (compassionate helping)

4. Additional task ^a^

5. Additional task ^a^

6. Mother-child interaction ^a^

7. Mother interview ^a^

8. Searching lost ball (instrumental helping)

9. Pain simulation (2) – mother or experimenter ^b^ (compassionate helping)

10. Out of reach pen (instrumental helping)

11. Additional task ^a^

12. Additional task ^a^

13. Sharing task.

*Notes.* ^a^ Tasks not included in the current report
^b^ Mother and experimenter distress simulations were counterbalanced.

**Order of tasks in home visits – 36 months**

1. Consent form

2. Mother-child interaction ^a^

3. Pain simulation – mother (compassionate helping)

Moving to a different room with the child and warm-up game

4. Keys placement (for searching instrumental task).

5. Additional task ^a^

6. Searching lost keys (instrumental helping).

7. Additional task ^a^

8. Broken doll sadness simulation – experimenter (compassionate helping).

9. Out of reach crayons (instrumental helping)

10. Additional task ^a^

11. Additional task ^a^

12. Additional task ^a^

13. Sharing task.

*Notes.* ^a^ Tasks not included in the current report

Table S1: *Spearman correlations between different subtypes of prosociality at each age*

| Sharing amount | Sharing stage | Compassionate total | Instrumental total |  |
| --- | --- | --- | --- | --- |
| .13 | .15^†^ | .35** | -- | Instrumental total |
| .19* | .13 | -- | .25** | Compassionate total |
| .96** | -- | .25** | .11 | Sharing stage |
| -- | .78** | .22** | .11 | Sharing amount |

^†^ p ≤ .10 * p < .05 ** p < .01 (all 2-tailed)

Table S2: *Partial correlations between different subtypes of prosociality at each age controlling for child's temperament*

| Sharing amount | Sharing stage | Compassionate total | Instrumental total |  |
| --- | --- | --- | --- | --- |
| .13 | .15 | .33** | -- | Instrumental total |
| .19* | .14 | -- | .25** | Compassionate total |
| .96** | -- | .22* | .10 | Sharing stage |
| -- | .77** | .19* | .11 | Sharing amount |

^†^ p ≤ .10 * p < .05 ** p < .01 (all significance tests are 2-tailed).

Notes: Partial spearman correlations presented, controlling for children's Positive Affectivity/Surgency reported at age 12 months. Correlations at 18 months are presented above the diagonal, and correlations at 36 months are presented below the diagonal.

Table S3: *Longitudinal links from 18 to 36 months, between different subtypes of prosocial behavior*

| *18 months:* | | | | *36 months:* |
| --- | --- | --- | --- | --- |
| Sharing amount | Sharing stage | Compassionate total | Instrumental total |  |
| .15 | .14 | .00 | .15^†^ | Instrumental total |
| -.11 | -.11 | .26** | .08 | Compassionate total |
| .14 | .16^†^ | .07 | .11 | Sharing stage |
| .19* | .19* | .21* | .19* | Sharing amount |

^†^ p ≤ .10 * p < .05 ** p < .01 (all significance tests are 2-tailed).

Notes: Spearman correlations are presented.

Table S4: *Longitudinal links from 18 to 36 months between different subtypes of prosocial behavior, controlling for child's temperament*

| *18 months:* | | | |  |
| --- | --- | --- | --- | --- |
| Sharing amount | Sharing stage | Compassionate total | Instrumental total |  |
|  |  |  |  | *36 months:* |
| .15 | .14 | -.01 | .14^†^ | Instrumental total |
| -.12 | -.12 | .23** | .03 | Compassionate total |
| .13 | .15^†^ | .04 | .07 | Sharing stage |
| .18* | .19* | .19* | .15† | Sharing amount |

^†^ p ≤ .10 * p < .05 ** p < .01 (all 2-tailed)

Notes: Partial correlations, controlling for children's Positive Affectivity/Surgency as reported by mothers at 12-months, are presented.

Table S5: The links between sharing stage and amount shared at 18 and 36 months

| 1. *18 months* | | | |  | 1. *36 months* | | | |
| --- | --- | --- | --- | --- | --- | --- | --- | --- |
|  | Single (n=23) | Some (n=16) | Most (n=9) |  |  | Single (n=28) | Some (n=51) | Most (n=21) |
| Compliance (n=22) | 15 (2.6) | 4 (-2.0) | 3 (-.8) |  | Compliance (n=63) | 20 (1.1) | 37 (2.0) | 6 (-3.7) |
| Cued (n=21) | 7 (-1.8) | 8 (-.6) | 6 (1.5) |  | Cued (n=16) | 6 (.9) | 9 (.5) | 1 (-1.6) |
| Spontaneous (n=5) | 1 (-1.3) | 4 (2.3) | 0 (-1.1) |  | Spontaneous (n=21) | 2 (-2.1) | 5 (-2.8) | 14 (5.8) |

*Notes.* 18 months: χ^2^(4) = 10.88, *p* = .028. 36 months: χ^2^(4) = 33.73, *p* < .001

Within each age, cells indicate the frequency (n) of each combination and, in brackets, the adjusted standardized residual, which reflects the difference between the expected and observed values as a Z score; all values greater than |1.96| are significant at p < .05 or less.
